# “*Molecular signature*” in blood of liver cancer diagnosed patients as a criterion for optimal therapy modalities

**DOI:** 10.1186/1878-5085-5-S1-A38

**Published:** 2014-02-11

**Authors:** Olga Golubnitschaja, Melanie Cebioglu, Kristina Yeghiazaryan, Claus C Pieper, Hans H Schild

**Affiliations:** 1 Department of Radiology, Rheinische Friedrich-Wilhelms-University of Bonn, Sigmund-Freud-Str. 25, Bonn 53105, Germany

## Scientific background and hypothesis

**Figure 1:**
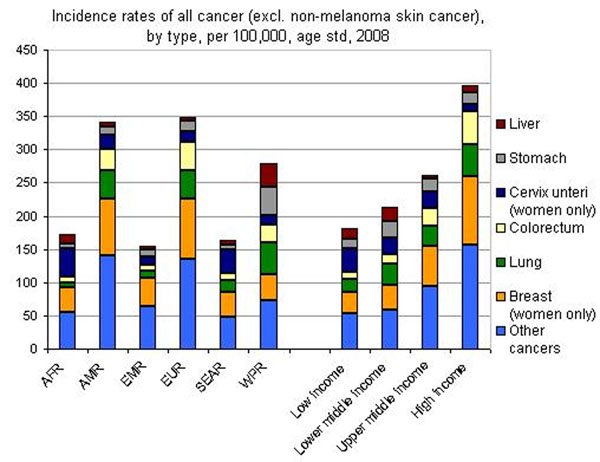
The WHO Regions for Europe and the Americas had the highest incidence of all types of cancer combined for both sexes. Countries in the WHO Eastern Mediterranean Region had the lowest incidence rates. Among the WHO Regions, the countries in the WHO Western Pacific Region had by far the highest incidence of stomach cancer and liver cancer. Men in the WHO Western Pacific Region had five times the rate of liver cancer of men in all other regions, except for Africa, where it was more than double the rate. Women in the WHO Western Pacific Region also had a considerably higher liver cancer incidence rate than women in other WHO regions [2].

In accordance with the statistical data reported by WHO, liver malignancies are far from the leading incidence among all cancers (Figure 1) but represent the 3^rd^ most fatal cancer type causing about 0,7 million deaths annually [1]. This discrepancy argues for a low efficacy of currently applied treatments. The consequent economical burden is unproportionally high, when the modest incidence of this cancer type is considered in the overall context of healthcare. Our hypothesis comprises possible reasons for the failure of long-term outcomes. On one side that may be the divergent origins of the liver cancer provoked by:

➣ hepatitis C

➣ cirrhosis of the liver abused with alcohol consumption

➣ heterogenic metabolic liver dysfunctions such as steatosis hepatis, etc.

➣ chronic inflammatory processes of different origin

➣ genetic predisposition

➣ toxic environment

all by its own way leading to primary liver carcinomas to be discriminated from completely different origin of the liver metastatic disease down-stream towards several cancer types primarily developed in colon, stomach, breast, prostate, pancreas, cervix, bronchus, skin, etc. On the other side this is the patient individuality as a kind of “complex biochemical reactor”, which strongly influences the disease onset and therapy outcomes. Hence, there are evident deficits in predictive molecular diagnostics and treatments tailored to the person to be considered for the field promotion.

## Material and methods

One hundred and six patients with primary liver malignancies and secondary liver metastatic disease have been recruited for a pilot study. The patients have been grouped according to a gender, type of primary tumour and an applied therapy (chemotherapy, TACE versus irradiation, SIRT). Blood samples have been taken before therapy, 1 day, 3 and 6 months after the therapy application. Each sample with full blood underwent immediate isolation of plasma and circulating leukocytes which got frozen and stored at -80°C for consequent analytical procedure. Individual as well as subgroup specific molecular profiles have been created using conventional technologies of clinical proteomics and sub-cellular imaging by quantitative “comet assay” analysis.

## Results interpretation and conclusions

Our results confirm the hypothesis concerning the divergent molecular profiles in corresponding subgroups. As proposed, all the above listed parameters strongly influence both the initial profiles before the therapy application and the reaction towards the applied therapy. Further, the molecular patterns are characteristic for each group created accordingly to the above nominated criteria. The most characteristic patterns have been identified for

➣ detoxification pathways

➣ microfilamental-network associated proteins

➣ stress proteome

➣ tissue remodelling enzymatic complex

➣ DNA fragmentation (comet patterns)

The principle of individual radiation sensitivity has been found to be the decisive parameter for choosing between SIRT and TACE.

## Recommendations

Large-scaled studies are highly recommended to validate preliminary results of the pilot study. Potential outcomes might be of highest value for developing of predictive diagnostics and personalisation of medical services in the cohort of patients with liver cancer.
